# Giant subcutaneous bronchogenic cyst in the intergluteal cleft region of an adult: a case report and literature review

**DOI:** 10.1186/s12880-022-00853-y

**Published:** 2022-07-16

**Authors:** Chuang-Yi Zheng, Shu-Yan Su, Rui-Bin Huang

**Affiliations:** 1 Department of Orthopedics, First Affiliated Hospital, Shantou University Medical College, Shantou, Guangdong People’s Republic of China; 2 Department of Radiology, First Affiliated Hospital, Shantou University Medical College, Shantou, 515041 Guangdong People’s Republic of China

**Keywords:** Bronchogenic cyst, Magnetic resonance imaging, Subcutaneous, Case report

## Abstract

**Background:**

Bronchogenic cysts (BCs) are generally detected in the mediastinum, along the tracheobronchial tree, or in the lung parenchyma. Subcutaneous BCs are rare, but, when found, are usually small (< 3 cm) and detected in children.

**Case presentation:**

In an unusual adult case, we treated a 52-year-old woman who presented with a mass in the left intergluteal cleft region. Ultrasonography showed a well-circumscribed hypoechoic lesion with posterior enhancement and internal echogenic foci within the mass. Color Doppler images showed no signals. Computed tomography showed the mass as a homogeneous, 6.8- × 6.3- × 5.1-cm soft tissue-attenuation lesion lodged in subcutaneous fatty tissue. Magnetic resonance imaging revealed a cystic lesion of similar dimensions with heterogeneous hyperintensity on both T1- and T2-weighted images. No contrast enhancement, solid components, or restricted diffusion foci were apparent. The cyst was completely excised, and histopathological evaluation indicated it was a BC. The patient’s recovery was uneventful.

**Conclusions:**

BCs should be considered in the differential diagnosis of all subcutaneous cystic masses, regardless of their location and size and the patient’s age.

## Background

A bronchogenic cyst (BC) is a congenital pulmonary anomaly resulting from abnormal budding of the tracheobronchial tree during embryological development [1]. Most BCs are located in the middle mediastinum near the trachea, the main bronchi, or lung parenchyma [1–4]. Subcutaneous BCs have been rarely reported [5–9]. When found, however, they are usually small (< 3 cm) and are most commonly located in the suprasternal notch, presternal area, neck, and scapula. Clinically, subcutaneous BCs generally appear shortly after birth or during early childhood. They are exceedingly rare in adults [8]. We found only five reported cases of subcutaneous BCs in people > 18 years of age in the English-language literature [5–9]. In addition, the presented case is only the second reported case of a giant cyst (> 6 cm) in an adult and the first such cyst located in subcutaneous fatty tissue of the intergluteal cleft region.

## Case presentation

A 52-year-old woman presented for evaluation of a painless mass in the left intergluteal cleft region. It had been present for 10 years but had enlarged significantly over the previous year. When the patient first noticed it a decade previously, the lesion had been the size of a peanut. The patient had no other relevant medical or trauma history. Physical examination revealed a 6-cm, partly compressible, superficial mass with no tenderness or associated skin changes. There was no visible fistulous opening or discharge from the lesion. Laboratory results—including complete blood count, biochemical blood tests, and tumor markers—were within their normal ranges.

**Fig. 1:**
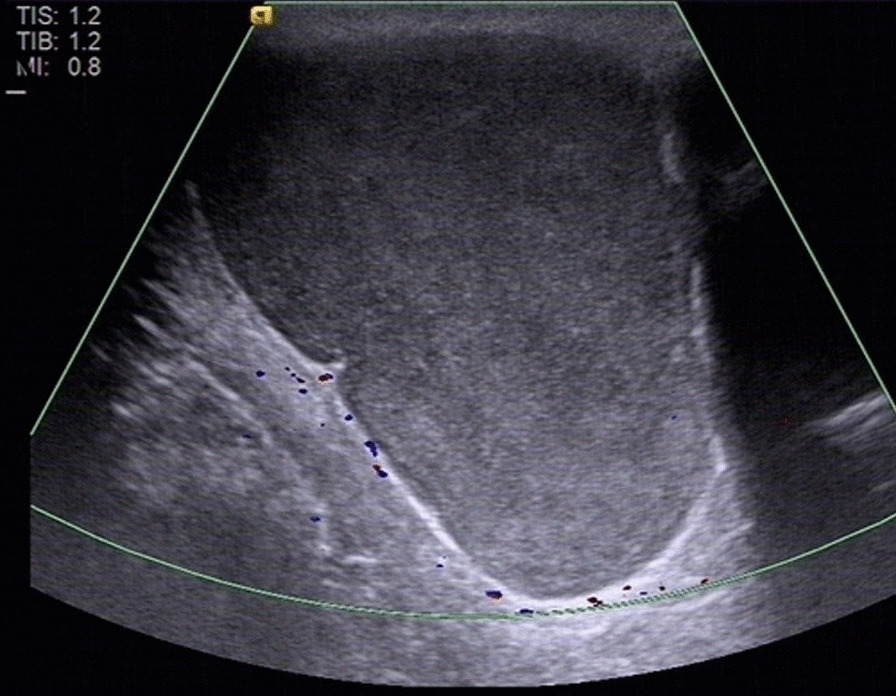
Ultrasonography shows a hypoechoic lesion with internal echogenic foci and posterior acoustic enhancement

**Fig. 2:**
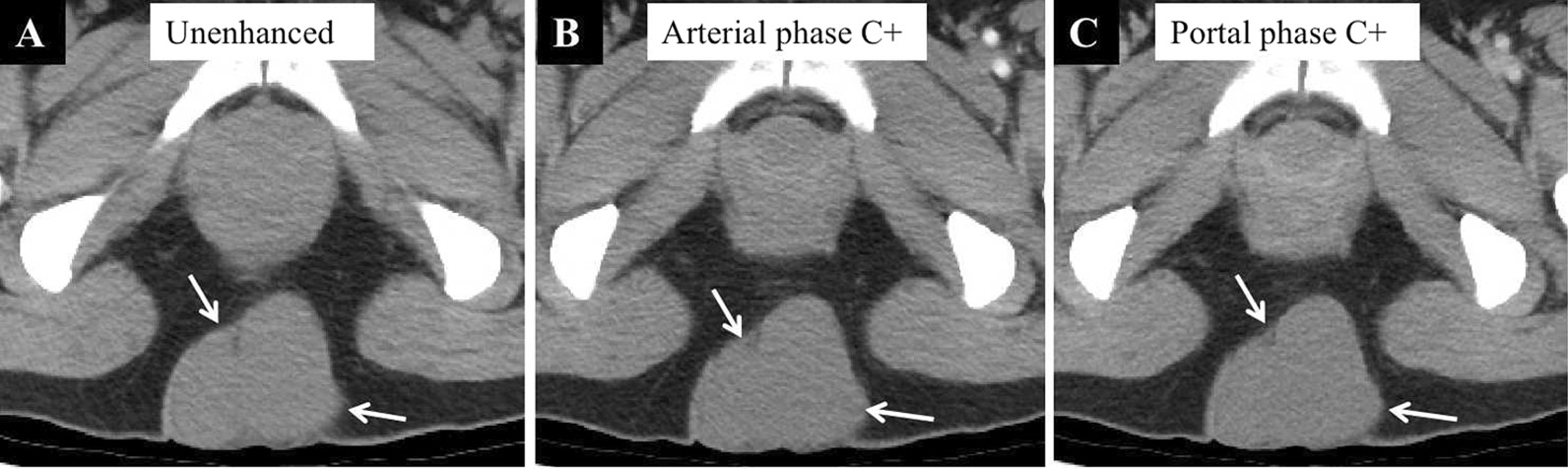
Preoperative computed tomography with and without contrast enhancement. Axial basal unenhanced image (**A**), arterial phase enhanced image (**B**) and portal phase enhanced image (**C**) show a lageniform, homogeneous, soft tissue-attenuation lesion (arrow) in the subcutaneous fatty tissue without calcification or post-contrast enhancement

**Fig. 3:**
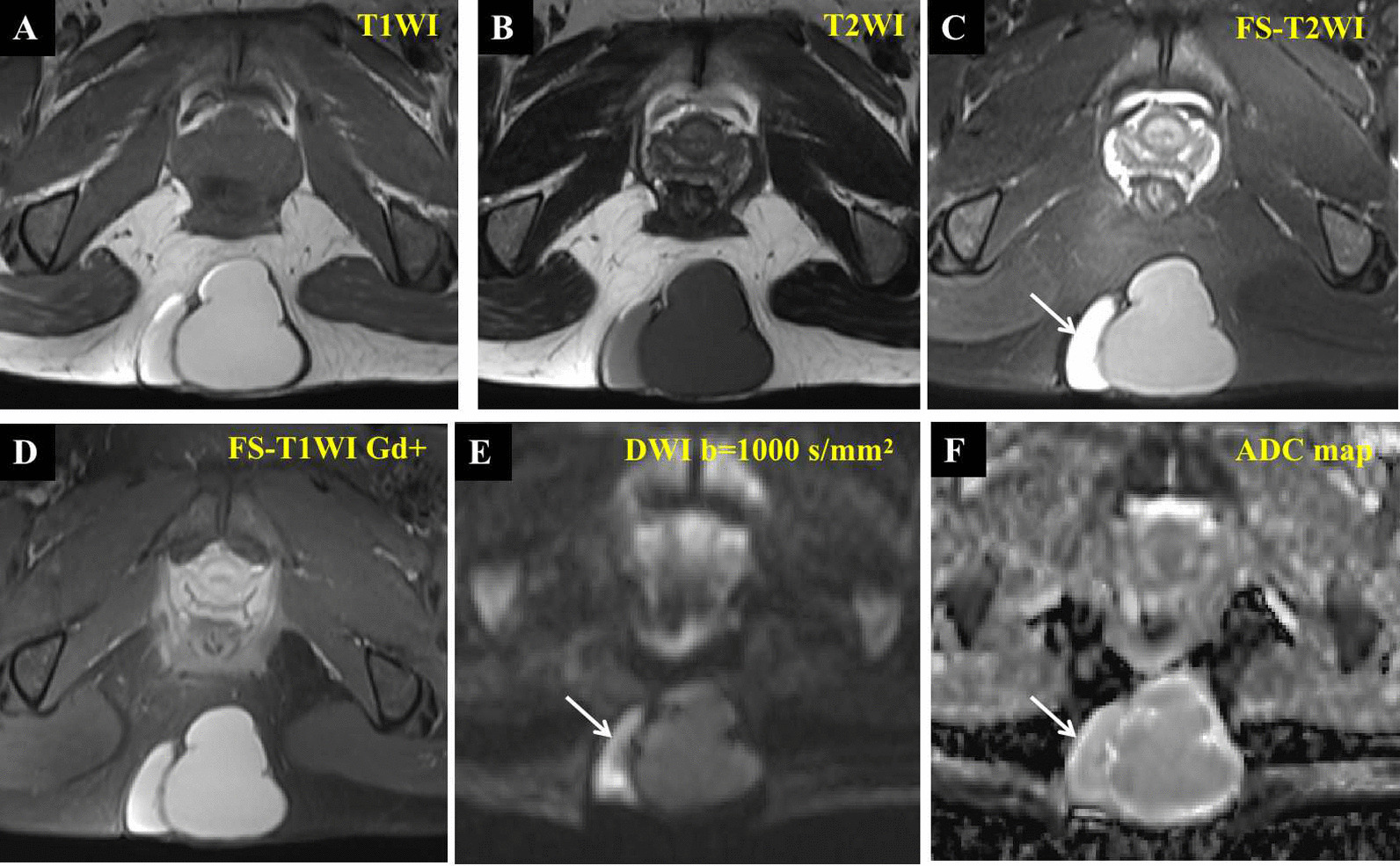
Preoperative magnetic resonance imaging. Axial T1 weighted image (**A**), T2 weighted image (**B**) and fat-sat T2 weighted image (**C**) show a cyst of similar dimensions with heterogeneous hyperintensity. Fat-sat post contrast T1 weighted image (**D**) reveals no contrast enhancement or evidence of solid components. (**E, F**) Axial diffusion-weighted images show signals of heterogeneous isointensity and hyperintensity (**E**, arrow) due to a T2-shine-through phenomenon (**C**, arrow) without restricted diffusion foci (**F**, arrow)

Ultrasonography showed a well-circumscribed hypoechoic lesion with posterior enhancement and internal echogenic foci. Color Doppler images showed no signal on the mass (Fig. 1). Subsequently, computed tomography (CT) showed a 6.8 × 6.3 × 5.1 cm, lageniform, homogeneous, soft tissue-attenuation lesion (41–52 HU) in the subcutaneous fatty tissue of the intergluteal cleft region (Fig. 2A–C). The lesion showed no internal calcification or post-contrast enhancement. Magnetic resonance imaging (MRI), performed to further characterize the mass, revealed a bilocular cystic lesion without contrast enhancement or solid components. The mass showed heterogeneous signal intensity comprising slight to marked hyperintensity on both T1- and T2-weighted images (Fig. 3A–C)—in contrast to adjacent muscle, which showed no contrast (Fig. 3D), solid components, or restricted diffusion foci (Fig. 3E, F). Based on these findings, the most likely diagnosis was a subcutaneous epidermoid cyst. Hence, the mass was surgically excised without postoperative complications.

**Fig. 4:**
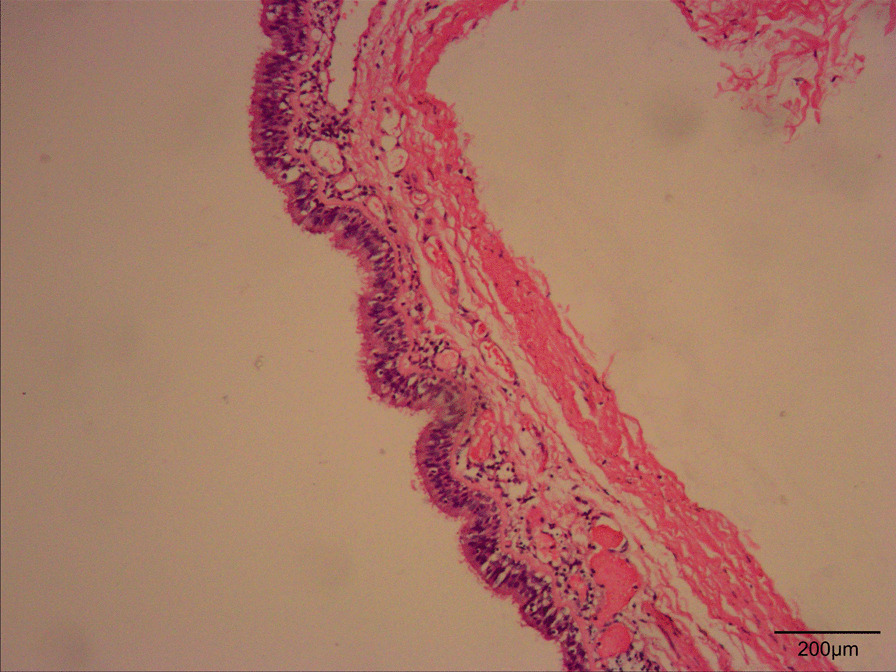
Histopathological examination was performed on an Olympus CX43 Biological Microscope and cellSens acquisition software. The Olympus CX43 settings were as follows: trinocular tube 2 with two widefield eyepieces for FN20, WHB10× objective in the light path, digital camera for microscope (DP22), and U-25LBD microscope polarizing filter (batch number 6,397,600). Histopathological examination with haematoxylin and eosin stain of the cystic lesion showed the characteristic ciliated pseudostratified columnar epithelium and cyst wall comprising fibrous tissue, inflammatory cells, and smooth muscle (original magnification ×100)

Macroscopically, it was a well-defined, gray-tan cystic mass that contained brownish mucous material. Histopathological examination of the resected cyst showed that it was lined with pseudostratified ciliated columnar epithelium, which was consistent with it being a BC. Hemorrhage, inflammatory cells, and fibrosis were present but no signs of malignancy (Fig. 4). The patient’s postoperative course was uneventful, and she was discharged from the hospital on postoperative day 4. Clinical follow-up and CT at 11 months showed no signs of recurrence.

## Discussion and conclusion

BCs are congenital foregut malformations caused by abnormal budding of the tracheobronchial tree during embryological development [1]. Histologically, BCs are typically lined with pseudostratified ciliated columnar epithelium. On occasion, however, they present with bronchial glands, cartilage, smooth muscle, and mucoid material. BCs have be found in both intrathoracic and extrathoracic locations [1, 2, 8, 10, 11]. More than 50% of BCs are located in the thoracic cavity [11]. Ectopic, extrathoracic BCs may occur in cutaneous or subcutaneous tissues, the neck, the scapular area, abdominal wall, or retroperitoneal area, among other sites [6, 8–11]. It remains unclear how these cysts reach such aberrant positions. Although subcutaneous BCs are rarely reported, their most common sites are the suprasternal notch, presternal area, neck, and scapula [5–8]. Our search showed no prior reports of subcutaneous BCs in the intergluteal cleft region. Our case appears to be unique.

Clinically, subcutaneous BCs have been found shortly after birth or during early childhood. More than 80 cutaneous or subcutaneous BCs have been reported in the English-language literature [8, 9]. Subcutaneous BCs in adults are extremely rare. A review of the reported data revealed only five such cases [5–9], which were all in men, and four of the five were in the presternal area. Only one of the BC lesions was > 6 cm.

The present report constitutes only the second reported case of a giant subcutaneous bronchogenic cyst (> 6 cm), and it is the first reported in an adult woman. The characteristics (e.g., age, sex, size, location, duration, imaging study, treatment, cystic fluid characteristics, outcomes) of the detected and reported subcutaneous BCs in adults, including the present case, are shown in Table 1.

**Table 1:** Summary of reported cases of subcutaneous bronchogenic cysts in adults

Author/year	Age (yrs)	Sex	Location	Size (cm)	Duration	Imaging studies	Surgical resection	Cystic fluid	Recurrence (follow-up)
Hameed et al. [7]/1993	19	M	Presternal area	–	1 year	Chest radiography	Total resection	Brownish fluid	–
Alar et al. [5]/2012	42	M	Presternal area	1.6	42 years	CT	Fine-needle aspiration	–	–
Moon et al. [9]/2017	18	M	Presternal area	3.3 × 1.7 × 3.1	–	Ultrasonography	Total resection	Whitish mucous material	–
Gaikwad et al. [6]/2006	34	M	Suprasternal notch	4.5 × 3.5 × 1.5	34 years	Ultrasonography	Total resection	White gelatinous material	–
Mangiameli et al. [8]/2020	20	M	Presternal area	3.7 × 1.4 × 6.5	1 year	CT, ultrasonography, MRI	Total resection	Whitish mucous material	No (6 months)
Present case	52	F	Intergluteal cleft region	6.8 × 6.3 × 5.1	10 years	CT, ultrasonography, MRI	Total resection	Brownish mucous material	No (11 months)

*yrs* years, *M* male, *F* female, *CT* computed tomography, *MRI* magnetic resonance imaging, — not available

Although ultrasonography, CT, and MRI are helpful for detecting a BC [1, 2, 4, 9, 12], MRI provides a better definition of the cyst itself. On ultrasonography, BCs frequently appear as anechoic, well-defined, rounded or elongated cysts and as internal echogenic foci with posterior acoustic enhancement in subcutaneous tissue [9, 12]. Likewise, BCs usually manifest as spherical masses of either water or soft-tissue attenuation/intensity. They are not enhanced on CT or MRI following intravenous administration of contrast agents [1, 2, 4]. These cysts comprise a mixture of water and proteinaceous mucus in different proportions, along with different calcium contents, which results in variable echoic features on ultrasonography, attenuation on CT, and intensity on MRI. In the present case, the lesion appeared as homogeneous high attenuation on CT and slight-to-marked hyperintensity on both T1- and T2-weighted images without restricted diffusion foci or contrast enhancement, which is likely due to the presence of methemoglobin, mucin, and proteins within the cyst. Note, when there is increased secretion of mucus from the cyst or secondary infection and bleeding occur, the lesion becomes more irregular with heterogeneous attenuation/intensity, making it more difficult to distinguish it from other diseases, as occurred in the present case. Sonographically guided fine-needle aspiration biopsy has been reported to be used for diagnostic purposes or ruling out a possible malignant cause (sarcoma) [8, 13]. However, this procedure should not serve as definitive treatment because cyst aspiration does not allow mucosal lining removal, which might cause rapid relapse or even malignant lesions transformation [14].

Because of their submucosal location and nonspecific imaging appearance, BCs are often misdiagnosed preoperatively as other subcutaneous cystic lesions. The most common growths in the differential diagnosis of subcutaneous BCs are pilonidal cysts, dermoid cysts, epidermoid cysts, and cystic hygromas [6, 8, 9, 11, 15]. *Pilonidal cysts* occur predominantly in males. Sonographically, these cysts usually involve the dermis and hypodermis and appear as saclike or bandlike structures that communicated with the base of widened hair follicles. Pathologically, the sinus where the hair enters is lined by stratified squamous epithelium with slight cornification [15]. *Dermoid cysts* are located around the hyoid bone and are echogenic owing to the presence of fat and osseo-dental structures [16]. *Epidermoid cysts* usually appear as well-circumscribed masses confined to the subcutaneous layer, with a high T2 signal and sometimes with low-signal-intensity debris with thin rim enhancement on contrast-enhanced T1-weighted images. They may also show diffuse restriction in diffusion-weighted imaging sequences due to liquid contents or disturbance in the directional orientation of keratin, or both [16, 17]. Of note, T2 shine-through effect due to long T2 values has been reported as a common pitfall in many cystic lesions, such as breast and ovarian cysts, which may appear bright on diffusion-weighted images causing false-positive findings [18], as also shown in the present case. *Cystic hygromas* usually show homogeneous watery density on CT scans [19]. Thus, in several cases, radiological identification of these cystic lesions is challenging, and a definitive diagnosis may depend on histopathological evidence.

Although BCs are asymptomatic and in many cases are discovered incidentally during medical checkups or workups for other diseases, complications associated with BCs—that is, infection, cyst rupture, bleeding, malignant transformation—have been reported [3, 17, 20, 21]. Surgical resection remains the most suitable treatment for BCs. The type of surgical resection depends on the location, the size of the lesion, and the surgeon’s expertise [8, 10, 11, 21].

In summary, BCs should be considered in the differential diagnosis of all subcutaneous cystic masses, regardless of their location and size and the patient’s age.

## Data Availability

All data generated or analyzed during this study are included in this published article.

## Abbreviations

BCs: Bronchogenic cysts
CT: Computed tomography
MRI: Magnetic resonance imaging
